# Occurrence of *Clostridium perfringens* in Shellfish

**DOI:** 10.3390/vetsci13010051

**Published:** 2026-01-07

**Authors:** Temitope C. Ekundayo, Frederick T. Tabit

**Affiliations:** Department of Life and Consumer Sciences, University of South Africa, Cnr Christiaan de Wet and Pioneer Ave, Florids, Private Bag X6, Roodepoort 1710, South Africa

**Keywords:** wild harvested shellfish, molluscs, *Crassostrea gigas*, *Ruditapes philippinarum*, *Mytilus galloprovincialis*, crustaceans, toxigenic clostridia, food safety, clams, mussels, oysters, shellfish beds

## Abstract

Background: *Clostridium perfringens* is an infectious agent of concern in wild/farmed shellfish. Hence, this study assessed shellfish-borne *Clostridium perfringens* (ShbCp) prevalence. Methods: A total of 1469 ShbCp from 2336 shellfish were modelled using hierarchical generalized linear and 1000-permutation-based-mixed-effects, meta-regression models. Results: The overall ShbCp prevalence was 54.12% (19.73–84.99) with a 32.02% (14.52–56.64) toxigenic rate and a higher estimate in 2020–2025 (41.01%, 17.00–70.23) versus 1970–2019 (20.01%, 4.49–57.08). Culture media significantly affect ShbCp recovery, with cooked meat medium and thioglycollate medium registering higher estimates (77% and 25.15%, respectively) than selective agars (<7%). The molluscans had a higher ShbCp rate (60.68%) than crustaceans (1.57%) and cephalopods (0.14%); oysters (85.97%) than mussels (71.81%), clams (50.38%), slug/snails (48.23%), scallops (16.24%), crabs (11.91%), shrimps (1.05%), and squids (0.42%); and *Crassostrea gigas* (89.27%) versus *Ruditapes philippinarum* (45.92%) versus *Mytilus galloprovincialis* (30.14%). ShbCp differed significantly by nations but not by continent with Spain (87.79%) having the highest rate, then China (47.01%), Japan (43.91%), the USA (10.44%), and Greece (0.00%); South America (51.36%), then Asia (44.77%), Europe (21.97%), and North America (10.44%). Sample size, growth medium, nation, and shellfish class significantly explained 27.58%, 72.30%, 67.52%, and 28.51% ($R^{2}$) variance in ShbCp prevalence, respectively. Conclusions: The present study estimated a high ShbCp prevalence, suggesting a significant public health risk. It recommends that *C. perfringens* should be incorporated as a supplemental indicator into shellfish safety/shellfish water quality monitoring alongside traditional indicators. Also, geographical data gaps from Africa, Latin America, the Middle East, and Oceania underline the need for national and global monitoring attention and priority on *C. perfringens* in shellfish/shellfish beds.

## Simple Summary

*Clostridium perfringens* constitutes an important foodborne risk in shellfish globally. The current study aimed at assessing the occurrence of *C. perfringens* in shellfish. The overall prevalence of *C. perfringens* in shellfish was 54.12%. There is also a 32.02% chance of contracting its toxigenic strains in shellfish. Generally, the mollusc shellfish had more *C. perfringens* contamination than the crustaceans. Common shellfish such as oysters (85.97%), mussels (71.81%), and clams (50.38%) harbour more *C. perfringens* contamination than other shellfish groups, also, *C. perfringens* contamination in shellfish is common in Spain (87.79%), China (47.01%), Japan (43.91%), and the USA (10.44%); and in South America (51.36%), Asia (44.77%), Europe (21.97%), and North America (10.44%). The presence of *C. perfringens* in shellfish, often consumed raw or undercooked, constitutes a significant public health risk. Thus, it will be beneficial to include *C. perfringens* in shellfish quality monitoring as an auxiliary quality indicator in addition to established indicators.

## 1. Introduction

Filter-feeding behaviours of shellfish enable them to bioconcentrate all types of pathogens, including *C. perfringens* from aquatic milieus. Natural contamination of mussels/oysters by *C. perfringens* has been reported at 23/24 sampling sites, with *E. coli* contamination only occurring at 2/24 sites in Madden and colleagues; this is because *C. perfringens* spores persist more effectively and remain viable in shellfish for longer periods than vegetative *E. coli* [1,2]. By contrast, *C. perfringens* contamination in crustaceans/finfish is usually lower [3,4]. However, *C. perfringens* contaminations, including toxigenic strains, have been found in cultured/wild molluscan shellfish, including clams, oysters, scallops, and slug/snails [3,5,6,7,8,9], crustacean shellfish such as crabs [10,11] and shrimp [4], and squid cephalopod shellfish [12]. Notwithstanding that crustacean/cephalopod shellfish could carry *C. perfringens*, molluscan shellfish such as bivalves serve as *C. perfringens*’ main reservoirs [13,14,15].

*C. perfringens* contamination in shellfish has been reported from multiple regions. For instance, in Asia, *C. perfringens* has been isolated from *Ruditapes philippinarum* clams [3,5], oysters [5,16], scallops [5,17], and snails [5]; in Europe from *Mytilus galloprovincialis* mussels [6,8,9] and *Crassostrea gigas* oysters [9]; in North America from *M. galloprovincialis*/*Mytilus* spp. [9] and *M. edulis* mussels [4], *C. gigas* oysters, and *Pandalus jordani* shrimps [4], *Corbicula fluminea*, *Tresus nuttalli*, *Macoma* spp., *Saxidomus nuttalli*, and *S. giganteus* clams [4,7]; and in South America from *Neohelice granulata*/*Cyrtograpsus angulatus* crabs [10] and *C. rhizophorae* oysters [18]. Most *C. perfringens* isolates reported in European, Asian, and American studies were enterotoxin-producing strains, especially type-F from clams and oysters [3]. *C. perfringens* isolated from asari clams in a Japanese study were genetically overlapped with *C. perfringens* from human sewage [3]. In addition, the U.S. Delaware Bay study documented that *Clostridium* spp. persists in cultured oysters and local waters throughout the summer period [19]. Despite the isolation of *C. perfringens* in shellfish, most shellfish safety standards only target *E. coli* and *Vibrio* as indicator pathogens, not *C. perfringens*. For instance, the official shellfish and seafood monitoring programmes primarily target *E. coli*, *Vibrio parahaemolyticus*, and *V. vulnificus* among potential hazards [20], but omit *C. perfringens*. The use of classical fecal indicators and *Vibrio* spp. in quality monitoring and regulation of shellfish quality means that *C. perfringens* is often under-monitored, under-surveyed, and under-reported in shellfish globally [3,20,21,22].

*C. perfringens* contamination of shellfish can occur at all production and distribution stages, including aquaculture/harvesting, or post-harvest handling and distribution. Its spores or vegetative cells can be taken up and concentrated from polluted waters during filter feeding. Furthermore, *C. perfringens* can in reality survive the regular post-harvest handling of shellfish at 4 °C [2]. Heat shock during cooking also enhances *C. perfringens* spore germination in various foods, and its cells can then grow under prolonged cooling and abusive temperature [23]. Following the ingestion of raw or insufficiently cooked *C. perfringens*-contaminated shellfish/foods, the spores sporulate in the human intestine, and vegetative cells grow, causing toxin-mediated gastroenteritis [24]. *C. perfringens* has the ability to produce several tissue-damaging enzymes and exotoxins, depending on the carriage of toxin genes (α, β, ε, ι, cpe, and netB) when it grows under favourable conditions [25,26]. For instance, *C. perfringens* is a major cause of foodborne illness in Japan, and its type-F strains alone have accounted for 20–40 foodborne outbreaks/year, often with thousands of cases [3]. Since all *C. perfringens* strains produce alpha toxin [27], its mere presence in shellfish poses food safety hazard concerns. However, to date, there is no routine or intentional tracking/monitoring of *C. perfringens* contamination in shellfish in many regions, which reflects a critical knowledge and data gap. In addition, its specific prevalence in some shellfish species is unknown. Since the role of *C. perfringens* in shellfish-borne illness and appropriate safety controls remain poorly defined, the present study aimed at assessing the occurrence of *C. perfringens* in shellfish globally, regionally, nationally, and by shellfish species. The current study is the first global meta-analysis on the prevalence of *C. perfringens* in shellfish, advancing knowledge beyond single-country studies. It provides a detailed analysis at multiple levels of granularity, including shellfish species, quantitative comparison of growth media, and explicit temporal trends.

## 2. Materials and Methods

### 2.1. Data and Study Strategy

Shellfish-borne *C. perfringens* (ShbCp) data were mined from Web of Science, Scopus, and PubMed from peer-reviewed primary research studies or surveys (articles) published in any language across all geographies from each repository’s inception until 8 September 2025, 14:42:13 GMT+0200. The combined keywords explored and adapted for each database as topical search was “(perfringens AND (seafood * OR shellfish * OR cuttlefish * OR squid * OR octopus * OR cattail * OR jellyfish * OR urchin OR barnacle * OR gooseneck * OR krill * OR lobster * OR crab * OR crawfish * OR crayfish * OR prawn * OR mollus * OR shrimp * OR periwinkle * OR whelk * OR limpet * OR conch * OR abalone * OR scallops OR cockle * OR clams OR mussel * OR oyster * OR bivalve *))”. The detailed database-specific search strings are provided in the Supplementary Materials. ShbCp contamination was defined as the presence of *C. perfringens* in any shellfish species detected via any standard microbiological assays, either by culture- or culture-independent-based techniques. The evidence-based synthesis is an integral part of the project approved by the College of Agriculture and Environmental Sciences_Health REC, University of South Africa, with the ethical clearance reference number 2025/CAES_HREC/6998. However, no new animal data were collected in this study.

### 2.2. Eligibility and Exclusion Standards

The ShbCp data from primary peer-reviewed research articles were selected (or otherwise excluded) based on the PRISMA 2021 protocol. Importantly, the full-texts of the study must be available in any language; the study descriptors (author, year), sample size, and *C. perfringens* outcome (incidence data, and optionally toxigenicity data) must be extractable; and the shellfish type/species (Sampletype), methodological details (e.g., isolation medium/confirmation method), and nation where the study was conducted must be specified. A study is deselected once one or more of the above-listed information is lacking. Only peer-reviewed research articles were included in this study.

### 2.3. Data Processing

Duplicate records in ShbCp data were merged in Zotero (version 7.0.13) with the merged collection exported and topically (title-abstract) screened in Excel version 2016 for data extraction by the first author. The selected records following topical screening were downloaded, read, and the research data were mined into extraction forms repetitiously in 2 independent trials by the first author. Language translation of non-English documents to English was achieved using Google Translate. Data contained in plots and graphs was mined using the Plotdigitizer software v3. Data validation was first performed using an equality test by comparing the two forms based on set theory ($\frac{form_{1}\;\cup \;form_{2}}{form_{1}\;\cap \;form_{2}}=1$), and subsequently checked by the coauthor for consistency. Any discrepancy/disagreement was discussed and resolved by the authors. The entire process is schematically represented in Figure S1.

### 2.4. Data Details

The extracted data include citation (author, year), sample size (N), shellfish type, *C. perfringens* incidence (P), growth medium, confirmation method, and nation. Optional and derivable data included toxigenicity, shellfish common name (as contained in the search strings), continent, class (*Cephalopoda*, *Crustacea*, and *Mollusca*), era (1970–2019, and 2020–2025), and period (1970–1990 and 2003–2025).

### 2.5. Data Synthesis

A total of 1469 ShbCp isolates, including 191 toxigenic strains, from 2336 shellfish collated from 19 studies (summarized in Table 1) out of 297 initial records were normalized using logit transformation [28]. Then, the transformed data were fitted to a hierarchical generalized linear model to compute the ShbCp overall/toxigenicity pooled effect size (prevalence) based on study-level random intercepts [29]. While maximum-likelihood methodology was used to explore non-combinability [30], we fitted Egger’s regression to assess publication bias [31] among the data sources. In addition, we fitted a disaggregated dataset of 37 shellfish species to mixed-effects subgroup models to identify heterogeneities utilizing shellfish type, name, confirmation method, class, medium, period, era, nation, and continent [32]. In addition, we fitted the disaggregated dataset to 1000-permutation-based univariate mixed-effects meta-regression models to assess the contributions of shellfish type, sample size, year, name, confirmation method, class, medium, period, era, nation, and continent to ShbCp prevalence (Table S1) [33,34]. The nature of the meta-regression inputs was either numerical or discrete [33,34]. All models were fitted using a maximum-likelihood link function. Finally, we evaluated and ranked the robustness of the various contributions using Akaike’s Information Criterion with a small sample correction (AICc). All models’ outputs were presented in forest plots/tables. The models were executed in R v. 4.5.1 (2025-06-13 ucrt) using metafor version 4.8-0 and meta version 8.2-0 packages.

## 3. Results

### 3.1. Overall and Toxigenic Prevalence of Shellfish-Borne C. perfringens

**Figure 1:**
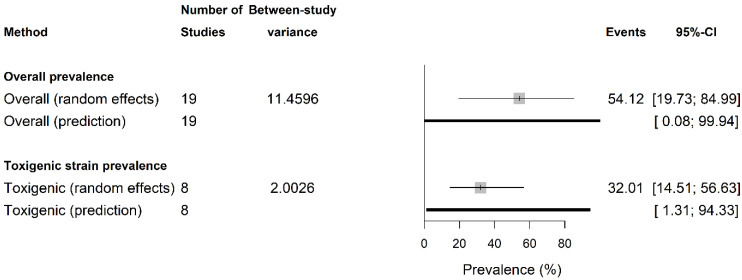
Prevalence of shellfish-borne *C. perfringens*.

The overall pooled ShbCp prevalence (A) and its toxigenic prevalence (B) are presented in Figure 1 (detail in Figure S2). The overall ShbCp prevalence was 54.12% (95% confidence intervals (CI): 19.73–84.99; prediction intervals (PI) = 0.08–99.94%; $I^{2}$ = 92.9%, 90.3–94.8, k = 19) and its toxigenic strain prevalence was 32.02% (14.52–56.64; PI = 1.32–94.33; $I^{2}$ = 92.7%, 87.9–95.6). There were no small-study effects in both estimates as presented by the Eggers’ test for overall ($\beta_{0}$ = 0.13, −3.19–3.45, *p* = 0.94) and toxigenic prevalence ($\beta_{0}$ = −5.82, −11.67–0.01, *p* = 0.098).

### 3.2. Temporal Pattern of Shellfish-Borne C. perfringens Prevalence

**Figure 2:**
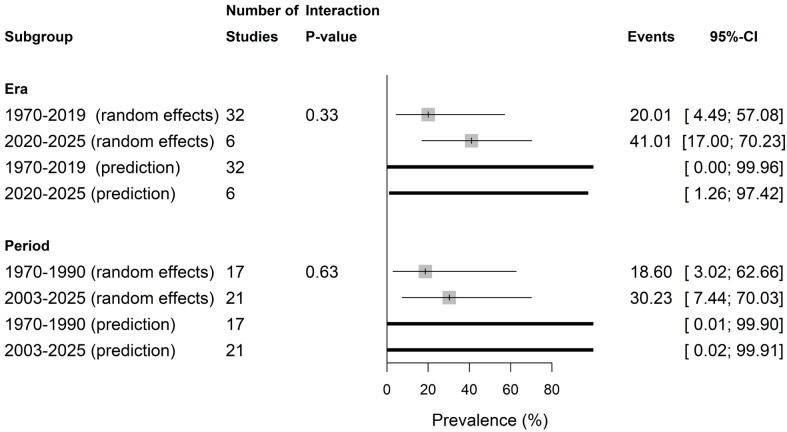
Prevalence of shellfish-borne *C. perfringens* by temporal patterns.

Figure 2 presents the temporal distinct patterns of ShbCp (detail in Figure S3). ShbCp prevalence was not significantly different (p=0.33) in rate in 2020–2025 (41.01%, 17.00–70.23; PI = 1–97.42%; $I^{2}$ = 92.2%) versus 1970–2019 (20.01%, 4.49–57.08, PI = 0.0–99.96%; $I^{2}$ = 82.1%). Also, ShbCp prevalence was higher in 2003–2025 (30.23%, 7.44–70.03; PI = 0.02–99.91%; $I^{2}$ = 78.4%) compared to 1970–1990 (18.60%, 3.02–62.66; PI = 0.01–99.90%; $I^{2}$ = 88.8%); however, the interactions were not significantly different (*p* = 0.63).

### 3.3. Shellfish-Borne C. perfringens Prevalence by Growth Media and Confirmation Method

**Figure 3:**
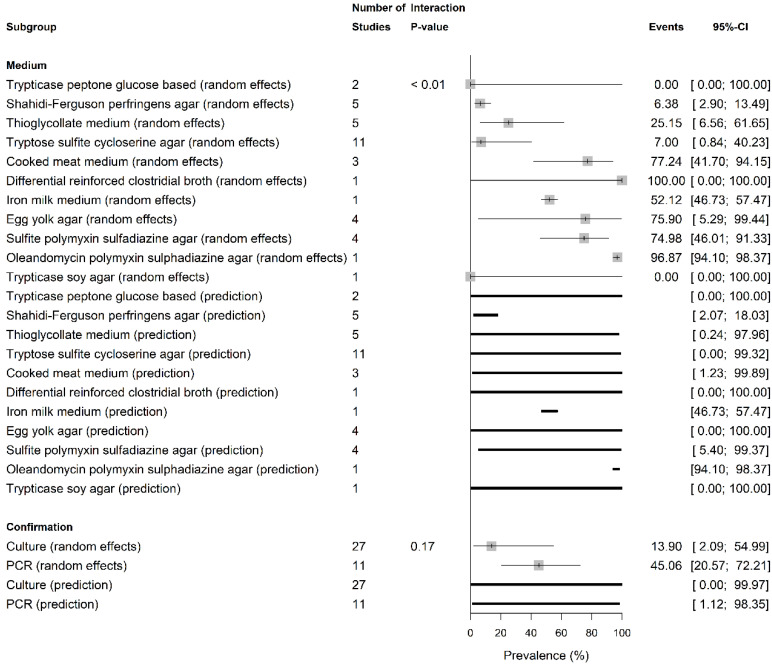
Pooled prevalence of shellfish-borne *C. perfringens* by growth media and confirmation method.

The pooled ShbCp prevalence according to growth media and *C. perfringens* confirmation methods is presented in Figure 3 and Figure S4. The pooled ShbCp prevalence was not significantly different between PCR (45.06%, 20.57–72.21; PI = 1.12–98.35%, k = 11; $I^{2}$ = 82.9%) and culture-based methods (13.90%, 2.06–54.99; PI = 0.0–99.97%, k = 27; $I^{2}$ = 88.2%) (*p* = 0.17; Figure 3). However, the pooled ShbCp prevalence was significantly different among growth media (*p* < 0.01), with the highest rate in cooked meat medium (77.24%, 41.70–94.15; PI = 1.23–99.89%; $I^{2}$ = 81.3%), followed by thioglycollate medium (25.15%, 6.65–61.65; PI = 0.24–97.96; $I^{2}$ = 91.2%), tryptose-sulfite-cycloserine agar (7.00%, 0.84–40.23; PI = 0.00–99.32; $I^{2}$ = 75.6%), Shahidi-Ferguson perfringens agar (6.38%, 2.90–16.49; PI = 0.00–99.32; $I^{2}$ = 0.0%), and trypticase peptone glucose-based agar (0%, 0.00–100.00; PI = 0.00–100.00; $I^{2}$ = 0.0%).

### 3.4. Shellfish-Borne C. perfringens Prevalence by Shellfish Scientific Name, Common Name, Scientific Class, Nation, and Continent

**Figure 4:**
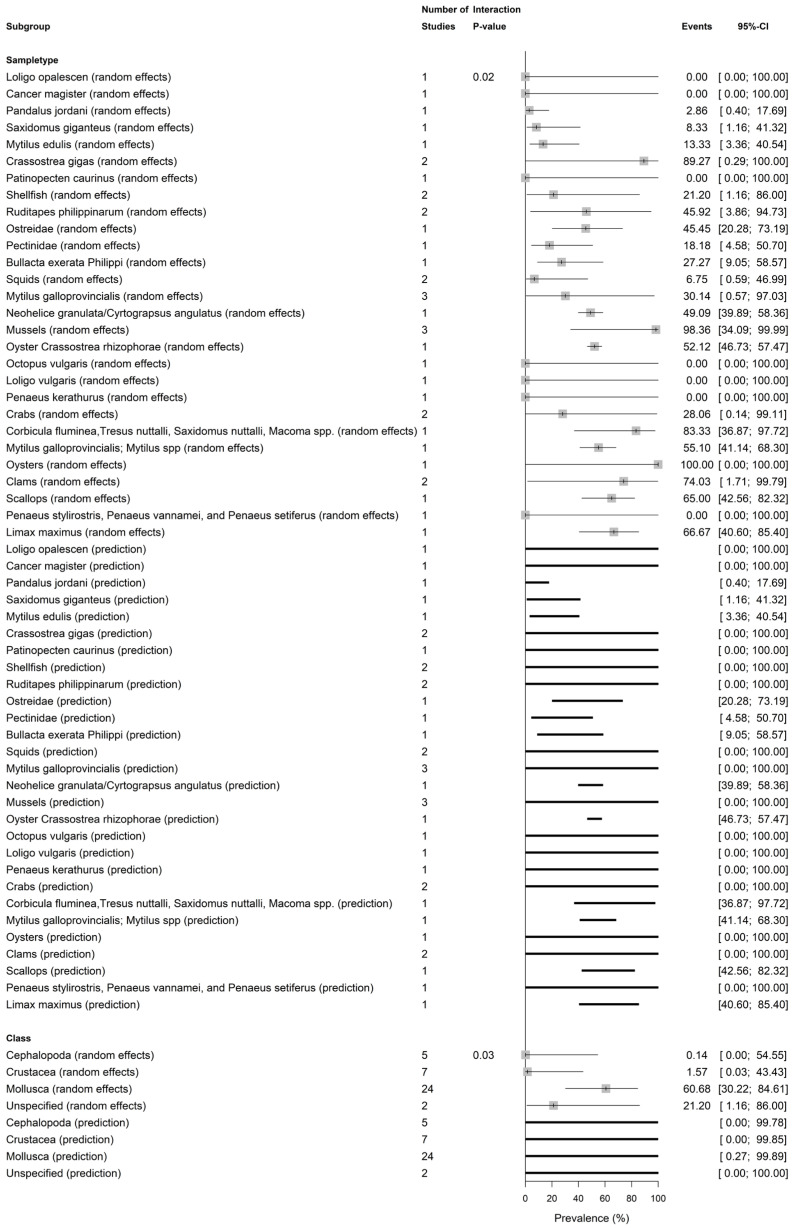
Shellfish-borne *C. perfringens* prevalence by shellfish scientific name and scientific class.

**Figure 5:**
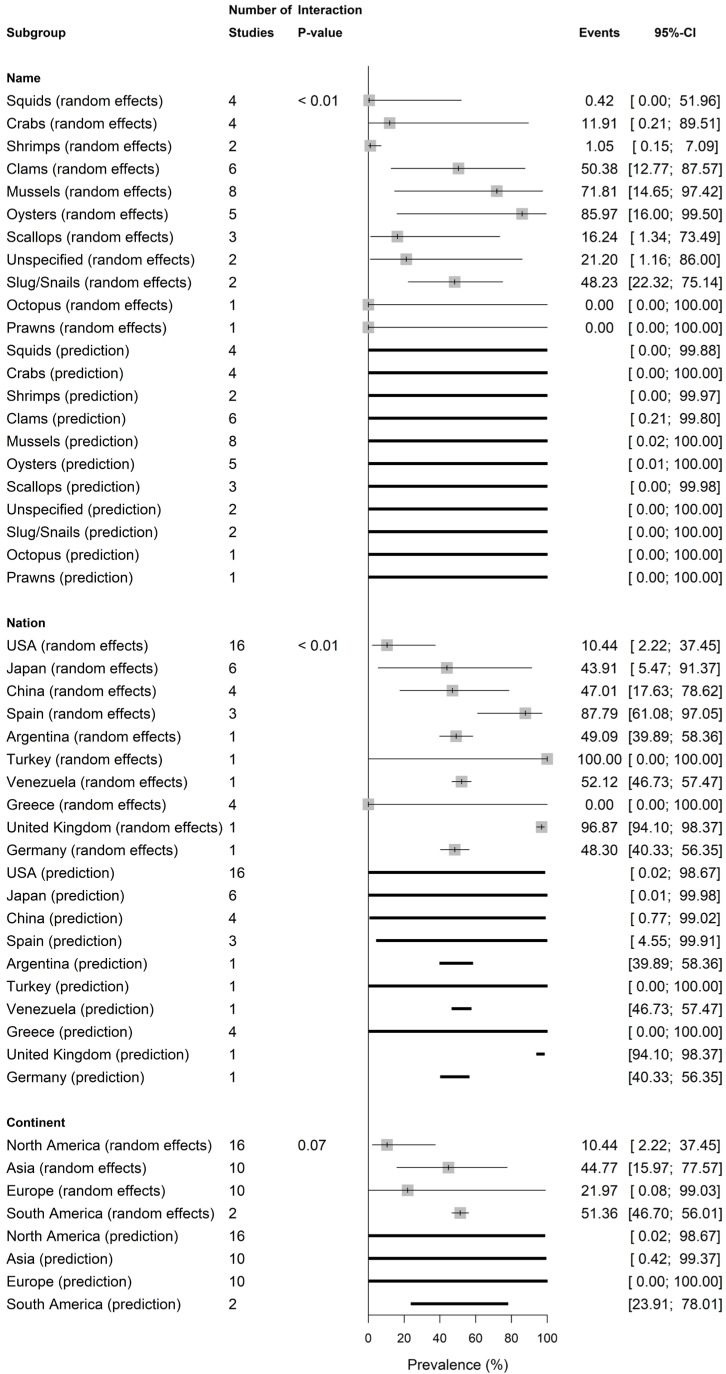
Shellfish-borne *C. perfringens* prevalence by shellfish common name, nation, and continent.

Figure 4, Figure 5 and Figure S5 present pooled ShbCp prevalence by shellfish scientific name, common name, scientific class, nation, and continent. The pooled ShbCp prevalence was significantly different (*p* = 0.02) by scientific name (Figure 4 and Figure S5A). The pooled prevalence was higher in *C. gigas* (89.27%, 0.29–100.00; $I^{2}$ = 0.0%), followed by *R. philippinarum* (45.92%, 3.86–94.73;${\;I}^{2}$ = 97.9%), and *M. galloprovincialis* (30.14%, 0.57–97.03, $I^{2}$ = 0.0%) (Figure 4 and Figure S5A).

ShbCp pooled prevalence was significantly different by common name (*p* = 0.001), with the highest rate in oysters (85.97%, 16.00–99.50; $I^{2}$ = 51.7%, k = 5), then mussels (71.81%, 14.65–97.42; $I^{2}$ = 93.2%, k = 8), clams (50.38%, 12.77–87.57; $I^{2}$ = 90.9%, k = 6), slugs/snails (48.23%, 22.32–75.14; $I^{2}$ = 72.9%, k = 2), scallops (16.24%, 1.34–73.49; $I^{2}$ = 63.1%, k = 3), crabs (11.91%, 0.21–89.51; $I^{2}$ = 72.0%, k = 4), shrimps (1.05%, 0.15–7.09; $I^{2}$ = 0.0%, k = 2), and squids (0.42%, 0.00–51.96; $I^{2}$ = 0.0%, k = 4) (Figure 5 and Figure S5B).

The pooled ShbCp prevalence was significantly different among shellfish classes (*p* = 0.0327), with the highest in *Mollusca* (60.68%, 30.22–84.61, $I^{2}$ = 88.3%, k = 24), *Crustacea* (1.57%, 0.03–43.43, $I^{2}$ = 74.6%, k = 7), and *Cephalopoda* (0.14%, 0.00–54.55, $I^{2}$ = 0.0%, k = 5) (Figure 4 and Figure S5C).

The pooled ShbCp prevalence was significantly different among nations (*p* < 0.01) with Spain (87.79%, 1.08–97.05, $I^{2}$ = 0.0%; k = 3) recording the highest rate, followed by China (47.01%, 17.63–78.62; $I^{2}$ = 86.3%, k = 4), Japan (43.91%, 5.47–91.37, $I^{2}$ = 89.0%; k = 6), the USA (10.44%, 2.22–37.45, $I^{2}$ = 74.4%; k = 16), and Greece (0.00%, 0.00–100.00, $I^{2}$ = 0.0%, k = 4) (Figure 5 and Figure S5D)

The pooled estimate among continents was highest in South America (51.36%, 46.70–56.01; $I^{2}$ = 0.0%, k = 2), then Asia (44.77%, 15.97–77.57; $I^{2}$ = 86.9%, k = 10), Europe (21.97%, 0.08–99.03; $I^{2}$ = 90.0%, k = 10), and North America (10.44%, 2.23–37.45; $I^{2}$ = 74.4%, k = 16) but were not significantly different (*p* = 0.07) (Figure 5 and Figure S5E).

### 3.5. Factors Influencing Shellfish-Borne C. perfringens Prevalence

The factors or variables influencing the prevalence of ShbCp are presented in Table 2. The factors that significantly influenced ShbCp prevalence in descending order were sample size (AICc = 180.44), growth medium (AICc = 181.97), nation (AICc = 182.33), and class of shellfish (AICc = 183.4). Sample size ranked as the best factor, with a regression weight of $\hat{\beta }=-1.32\pm 0.45$ and a significant interaction term ($F_{1,36}$ = 10.23, *p* = 0.002), but only accounted for 27.58% ($R^{2}$) of the true differences in ShbCp prevalence. The growth medium ranked in the second position with a $\hat{\beta }=1.08\pm 1.02$, a significant interaction term ($F_{10,27}$ = 3.94, *p* = 0.001), and accounted for 72.30% ($R^{2}$) of the true ShbCp prevalence differences. Among the growth media, differential reinforced clostridial broth (6.01±2.52, p=0.018), Shahidi-Ferguson perfringens agar (−3.64±1.34, p=0.007), trypticase peptone glucose-based agar (−4.74±1.93, p=0.019), trypticase soy agar (−5.88±2.52, p=0.030), and tryptose-sulfite-cycloserine agar (−2.42±1.18, p=0.030) significantly moderated ShbCp prevalence compared to others. Nation and shellfish class ranked as the third and fourth significant factors influencing ShbCp prevalence, with a $\hat{\beta }=-0.04\pm 1.64$ and $\hat{\beta }=-3.33\pm 1.18$, and robust interaction terms of $F_{9,28}$ = 3.79 (*p* = 0.001) and $F_{3,34}$ = 3.72 (*p* = 0.014), respectively. While nation accounted for 67.52% ($R^{2}$) of the true variance, shellfish class accounted for 28.51 ($R^{2}$) variance in the true ShbCp prevalence.

## 4. Discussion

The current study synthesized ShbCp prevalence at granular levels, including shellfish species, common names, class, nation, and continent, to provoke level-specific intervention in preventing shellfish safety risk and illness for the first time.

The study estimated an overall pooled ShbCp prevalence of 54.12% with a pooled toxigenic prevalence of 32.02%. These findings imply that shellfish/shellfish beds harbour *C. perfringens* as an indication of fecal contamination. The finding aligns with Madden and co-workers, who detected *C. perfringens* spores in nearly all wild oysters/mussels/beds they studied, even when *E. coli* was not detected [2]. The high prevalence of ShbCp observed has important implications for shellfish safety, public health, and waste/water management. First, *C. perfringens* spores’ exceptional survival ability in shellfish means their accumulation could indicate occult sewage pollution when other bacteria, such as *E. coli*, fail [2,3,21,39]. Secondly, the high occurrence of ShbCp signals a public health red flag for shellfish consumption since all *C. perfringens* produce alpha toxin [27]. Thus, the bare presence of *C. perfringens* or its spores at any level in shellfish constitutes a potential non-trivial risk if contaminated shellfish are eaten raw/undercooked or subjected to poor storage/handling; *C. perfringens*’ spores are heat-resistant and survive standard cooking/depuration processes [2]. Hence, shellfish deemed “clean” and “safe” by traditional *E. coli*/coliform standards may still harbour *C. perfringens* spores. *C. perfringens* causes human foodborne gastroenteritis and is responsible for an estimated $2.5\;\times 10^{5}$ cases of food poisoning annually in the United States alone [40]. Also, *C. perfringens* (type F) ranked as the second leading cause of bacterial food poisoning in Japan with ~3000 cases and 20–40 outbreaks annually [3,41]. While perfringens infections are typically self-limiting, they can be more severe among the very young or the elderly [40].

The high prevalence of ShbCp as found in this study, especially in oysters (≈86% prevalence) and mussels (≈72%), raises seafood safety concerns [2,14], and indicates environmental contamination, requiring urgent action from a regulatory standpoint. Currently, shellfish sanitation/safety criteria rely on *E. coli*, *Salmonella*, enterococci, or *Vibrio* [42,43], yet *C. perfringens* (type F) is increasingly recognized and advocated as a useful “alternative” fecal indicator of sewage pollution in waters since its spores have a longer decay time than other indicators [3,21]. The use of unsafe reclaimed water should thus be avoided for growing shellfish, as this practice has been linked to disease outbreaks in both developed and developing countries [44]. Hence, shellfish/water regulators should include *C. perfringens* spore counts (or the enterotoxin gene) as an auxiliary criterion for shellfish growing waters classification and in assessing depuration.

The temporal pattern of ShbCp prevalence highlights a higher rate between 2020 and 2025 (41.01%) versus 1970–2019 (20.01%), and between 2003 and 2025 (30.23%) compared to 1970–1990 (18.60%), respectively, these observations might reflect an increasing interest in ShbCp monitoring, true environmental changes such as altered wastewater discharges/flows into shellfish beds and shifts in shellfish harvest practices and/or the use of more *C. perfringens*-sensitive detection protocols.

The current data unveiled ShbCp prevalence differences according to growth media used and *C. perfringens* confirmation methods, with high heterogeneity levels ($I^{2}$ ≈ 90%) and wilder PIs. The $I^{2}\geq 75$ indicated significant heterogeneity [30]. Detection method (culture (13.90%) vs. PCR (45.06%)) did not significantly change the pooled ShbCp prevalence, but culture media significantly altered the pooled ShbCp prevalence. Rich media such as cooked meat medium and thioglycollate medium yielded higher *C. perfringens* recovery rates (77% and 25.15%, respectively) than selective agars (<7%), indicating that the methodology applied may strongly influence the reported prevalence of ShbCp.

While the estimate was not unduly influenced by small-study effects, as Egger’s test found no bias, which strengthens confidence in the results, uneven data coverage remains a major shortcoming that cautions against generalizing the global estimate.

ShbCp prevalence varied among shellfish species and class in this study. It is unsurprising that the current findings significantly highlight differences in ShbCp prevalence among shellfish species and shellfish classes, with molluscan shellfish (oysters/mussels/clams: 60.68%) registering higher rates than crustaceans (1.57%) and cephalopods (0.14%). Specifically, oysters (85.97%) had higher ShbCp than mussels (71.81%), clams (50.38%), slug/snails (48.23%), scallops (16.24%), crabs (11.91%), shrimps (1.05%), and squids (0.42%). Bivalve species such as *C. gigas* (89.27%), *R. philippinarum* (45.92%), and *M. galloprovincialis* (30.14%) had high *C. perfringens* contamination. This is generally expected as the finding agrees with the biology of molluscan shellfish, which are filter-feeders that selectively accumulate bacteria, spores, viruses, and particles from surrounding waters, unlike the lower/non-filter feeding crustaceans and squid that accumulate fewer particles (bacteria, spores, viruses, particles, and spores) from their surroundings [13,14]. Thus, the ShbCp hazard is chiefly a problem associated with filter-feeding shellfish that are consumed raw or undercooked [15]. As such, shellfish consumer advisories should emphasize effective cooking or thorough purification of raw molluscan shellfish, especially clams, oysters, and mussels prior to consumption. In addition, monitoring of anaerobic spore-formers, including *C. perfringens*, in shellfish beds should be prioritized as a supplemental indicator by environmental agencies, particularly in areas near sewage outlets.

ShbCp prevalence differed significantly by nations but not significantly by continent. While Spain (87.79%) had the highest ShbCp pooled rate, followed by China (47.01%), Japan (43.91%), the USA (10.44%), and Greece (0.00%). In terms of nation, the highest pooled ShbCp prevalence was observed in South America (51.36%), followed by Asia (44.77%), Europe (21.97%), and North America (10.44%). However, ShbCp data showed notable geographical sparsity and gaps, with most data largely coming from Europe, parts of Asia (China, Japan), and North/South America. Strikingly, there were no ShbCp data from Africa, Latin America, the Middle East, or Oceania in the current investigation. These obvious differences in geographical ShbCp data distribution could likely reflect differences in *C. perfringens* monitoring and reporting in shellfish and shellfish beds, differences in types of shellfish consumed, shellfish safety sanitation intensity, and quality indicator (*E. coli*, fecal) prioritization in the regions [42,43]. The regions lacking ShbCp data may have *C. perfringens* contamination issues in shellfish, but without regular monitoring and surveillance, it is impossible to assess *C. perfringens* occurrence or risk in such regions. Additionally, the variation in ShbCp data by geography could also reflect differences in wastewater discharge, environmental management, sanitation, and monitoring for *C. perfringens* and anaerobic bacteria across regions. For example, intensive aquaculture and heavy discharge of sewage into waterbodies have been linked to high rates of *Clostridioides difficile* contamination in Italy [13,45,46], whereas ShbCp and clostridia remain unstudied in shellfish and shellfish beds in Africa, Latin America, the Middle East, Eastern Europe, many parts of Asia, and Oceania. This generally agrees with the longstanding conclusion of the National Academies that “only rudimentary epidemiological data” exist for many seafood hazards, especially in under-resourced areas [15].

The factors influencing ShbCp prevalence significantly identified in this study were sample size, growth medium, nation, and shellfish class, which explained 27.58% ($R^{2}$), 72.30% ($R^{2}$), 67.52% ($R^{2}$), and 28.51 ($R^{2}$) variance in the true ShbCp prevalence, respectively. Sample size and culture medium largely governed and dominated ShbCp prevalence variation, suggesting the need for a standardized sampling (sample size) and culture protocols in assessing shellfish safety for comparability [47]. For instance, sample sizes can significantly affect ShbCp prevalence estimates, with smaller studies tending to yield spuriously high rates (Figure 1 and Figure S2). Geography and shellfish species differences are other factors that influenced ShbCp prevalence. The molluscan shellfish showed more contamination than crustaceans or cephalopods (Figure 4 and Figure S5). This underscores regional differences in surveillance intensity and environmental sanitation (e.g., sewage inputs) [3,48].

The current synthesis has important strengths and limitations that are worth highlighting. The strengths of the study consisted in offering a robust estimate of ShbCp prevalence at granular levels such as shellfish species, types/class, nation, and continent for the first time to enable targeted, level-specific interventions for promoting shellfish safety, which cannot be achieved by single studies or studies limited to one country. The estimate of the ShbCp toxigenic data in this study is invaluable from a public health context. Furthermore, the study identified key factors (methods, sample size, growth medium choice) moderating ShbCp prevalence to enhance and optimize targeted improvement in surveillance activities. Additionally, the lack of small-study bias, as indicated by Eggers’ test, lends confidence to the pooled ShbCp estimates in this study.

Nonetheless, the high $I^{2}$ and wide PIs that accompany the estimates indicate that local conditions dominate and affect ShbCp outcomes; thus, cautious interpretation should be considered. Another limitation of the study is methodological differences across ShbCp data, which could result in ShbCp rate under- or over-estimation, depending on the protocols adopted, ultimately weakening comparability. The choice of growth and enrichment media, specifically, clearly affects the detection of ShbCp, with cooked meat medium achieving a higher detection rate than standard agar. The implementation of random-effects models, prediction intervals, and bias tests, however, improved rate comparability. Finally, the non-standardization of sampling/sample sizes, the relatively small number of ShbCp data sources (k = 19), and their non-uniform geographic spread limit global generalization of estimates. Many locations, including major shellfish-producing regions in Africa, South America, Oceania, Eastern Europe, and parts of Asia, are not represented. Until these gaps are filled, the global patterns of ShbCp prevalence must be interpreted with caution. Despite the uneven geographic ShbCp data representation, the absence of ShbCp data from regions lacking data should not be interpreted as evidence of the absence of *C. perfringens* contamination in shellfish from those regions. Finally, while no publication bias was detected, it remains possible that negative findings (no *C. perfringens* found) are underreported or unpublished in the literature.

## 5. Conclusions and Future Action

In summary, the present study estimated a high prevalence of ShbCp, suggesting significant public health implications. It recommends that *C. perfringens* should be incorporated as a supplemental indicator into shellfish safety/shellfish water quality monitoring alongside traditional indicators. Since *C. perfringens* spores are effective proxies for protozoan parasites because of their shared resistance to inactivation treatments [49,50], their assessment would indirectly prevent contamination risk from other hardy pathogens (e.g., *Cryptosporidium*) in shellfish/shellfish beds. Additionally, shellfish growers and wild shellfish harvesters should ensure an effective depuration and carry out routine *C. perfringens* spore testing for every harvest. Furthermore, countries with large aquaculture industries and scarce water resources should invest in advanced wastewater treatment technologies that ensure *C. perfringens* spores and other hardy pathogens are inactivated before treated wastewater is reused in shellfish growing to ensure shellfish food safety. The current ShbCp data gap should be filled as a priority, especially in developing regions, to provide baseline data that would inform ShbCp risk and illness assessments.

## Figures and Tables

**Table 1:** Summary of shellfish-borne *Clostridium perfringens* data sources.

No.	Citation	Sample Size	Incidence	Toxigenicity	Shellfish	Confirmation	Medium	Nation
1	[27]	65	44	44	Molluscs (shellfish)	PCR	Thioglycollate medium	Japan
2	[5]	74	46	46	Molluscs (clams/oysters/snails)	PCR	Tryptose sulfite cycloserine agar (TSCA)/*C. perfringens* chromogenic medium	China
3	[3]	76	7	7	Molluscs (clams)	PCR	Thioglycolate broth	Japan
4	[12]	36	2	2	Molluscs (shellfish/squid)	PCR	Tryptose sulfite cycloserine agar	USA
5	[6]	15	13		Molluscs (mussels)	Culture	ISO 7937:2004 (TSCA)	Spain
6	[10]	110	54	54	Crustacean (crabs)	PCR	Cooked meat medium	Argentina
7	[8]	600	600		Molluscs (mussels)	Culture	Differential reinforced clostridial broth	Turkey
8	[18]	330	172		Molluscs (oyster)	Culture	Iron milk medium	Venezuela
9	[35]	155	0		Crustacean (prawns)/Molluscs (squid/octopus/mussels)	Culture	ISO 7937:1997 (SCA/TSCA)	Greece
10	[7]	59	32		Molluscs (clams/mussels/Crustacean (crabs)	Culture	Egg yolk agar	USA
11	[9]	60	48		Molluscs (mussels/oysters)	PCR	Sulfitep-olymyxin-sulfadiazine agar	Spain
12	[16]	41	41	9	Molluscs (oysters)	Culture	Egg yolk agar	Japan
13	[17]	40	15	4	Molluscs (squid/scallop/clams)	Culture	Thioglycollate medium	Japan
14	[2]	288	279		Molluscs (mussels)	Culture	Oleandomycin-polymixin-sulfadiazine-perfringens agar	UK
15	[4]	132	6		Crustacean (Crab/shrimp)/Molluscs (oyster/clam/scallop/mussel/squid)	Culture	Shahidi-Ferguson perfringens agar	USA
16	[36]	60	0		Crustacean (shrimps)	Culture	Trypticase soy agar	USA
17	[11]	33	29	25	Crustacean (crab)/Molluscs (clams)	Culture	Cooked meat medium/fluid thioglycollate medium	USA
18	[37]	147	71		Molluscs (mussels)	Culture	Sulfite polymyxin sulfadiazine agar	Germany
19	[38]	15	10		Molluscs (slug)	Culture	Sulfite polymyxin sulfadiazine agar	USA

**Table 2:** Meta-regression outcomes for factor influencing *shellfish-borne C. perfringens* prevalence.

Model	$\hat{\beta }$ ± SE	R^2^	AICc	Test of Moderators
Sample size	−1.32±0.45	27.58	180.44	$F_{1,36}=10.23,\;p=0.002$
Medium	1.08±1.02	72.30	181.97	$F_{10,27}=3.94,\;p=0.001$
Nation	−0.04±1.64	67.52	182.33	$F_{9,28}=3.79,\;p=0.001$
Class	−3.33±1.18	28.51	183.74	$F_{3,34}=3.72,\;p=0.014$
Confirmation	−0.80±0.53	0.76	190.30	$F_{1,36}=0.35,\;p=0.55$
Year	−24.97±51.71	0.55	190.50	$F_{1,36}=0.22,\;p=0.62$
Continent	−0.19±0.83	10.83	192.93	$F_{3,34}=0.93,\;p=0.40$
Name	−0.17±0.99	44.88	201.30	$F_{10,27}=1.56,\;p=0.12$
Shellfish type	−0.98±2.75	73.53	418.41	$F_{27,10}=0.60,\;p=0.87$

$\hat{\beta }$ ± SE = predicted effect size when x = 0 (regression coefficient)/standard error; R^2^ = coefficient of determination; df1 and df2 are degrees of freedom and Akaike′s Information Criterion with a small sample correction (AICc).

## Data Availability

The original contributions presented in this study are included in the article/Supplementary Materials. Further inquiries can be directed to the corresponding author.

## Supplementary Materials

The following supporting information can be downloaded at: https://www.mdpi.com/article/10.3390/vetsci13010051/s1, Figure S1: Flow diagram for processing data sources on shellfish-borne *C. perfringens*; Figure S2: Detailed prevalence of shellfish-borne *C. perfringens*; Figure S3: Detailed prevalence of shellfish-borne *C. perfringens* by temporal patterns; Figure S4: Detailed pooled prevalence of shellfish-borne *C. perfringens* by growth media and confirmation method; Figure S5: Detailed shellfish-borne *C. perfringens* prevalence by shellfish scientific names, common name, scientific class, nation, and continent. Table S1: Full coefficients of the various variables in the meta-regression models.
