# Supplementary material for: Occurrence of Clostridium perfringens in Shellfish
Source: Vet Sci. 2026 Jan 7;13(1):51. doi: 10.3390/vetsci13010051 (PMC12846457; doi:10.3390/vetsci13010051)
Supplement: Supplementary file 1 [file vetsci-13-00051-s001.zip › vetsci-4014180-supplementary.pdf]

Detail search strategies

Web of Science

TS=(perfringens* AND (seafood* OR shellfish* OR bivalve* OR oyster* OR mussel* OR clams OR cockle* OR scallops OR abalone* OR conch* OR limpet* OR whelk* OR periwinkle* OR shrimp* OR mollus* OR prawn* OR crab* OR lobster* OR krill* OR gooseneck* OR barnacle* OR urchin OR jellyfish* OR cattail* OR octopus* OR squid* OR cuttlefish*)) AND DT=(Article) Timespan: 1985-01-01 to 2025-08-07 Date Run: Mon Sep 08 2025 14:42:13 GMT+0200 (South Africa Standard Time)

Scopus

(TITLE-ABS-KEY((perfringens* AND (seafood* OR shellfish* OR bivalve* OR oyster* OR mussel* OR clams OR cockle* OR scallops OR abalone* OR conch* OR limpet* OR whelk* OR periwinkle* OR shrimp* OR mollus* OR prawn* OR crab* OR lobster* OR krill* OR gooseneck* OR barnacle* OR urchin OR jellyfish* OR cattail* OR octopus* OR squid* OR cuttlefish*))) AND PUBYEAR < 2026 AND (LIMIT-TO (DOCTYPE,"ar")))

PubMed

(perfringens[tiab] AND (seafood*[tiab] OR shellfish*[tiab] OR bivalve*[tiab] OR oyster*[tiab] OR mussel*[tiab] OR clams[tiab] OR cockle*[tiab] OR scallops[tiab] OR abalone*[tiab] OR conch*[tiab] OR limpet*[tiab] OR whelk*[tiab] OR periwinkle*[tiab] OR shrimp*[tiab] OR mollus*[tiab] OR prawn*[tiab] OR crab*[tiab] OR lobster*[tiab] OR krill*[tiab] OR gooseneck*[tiab] OR barnacle*[tiab] OR 'sea urchin'[tiab] OR jellyfish*[tiab] OR cattail*[tiab] OR octopus*[tiab] OR squid*[tiab] OR cuttlefish*[tiab])) AND 1900/01/01:2025/07/07[dp] NOT (review[Publication Type] OR systematic review[Publication Type] OR meta-analysis[Publication Type] OR retracted publication[Publication Type])

Supplementary results

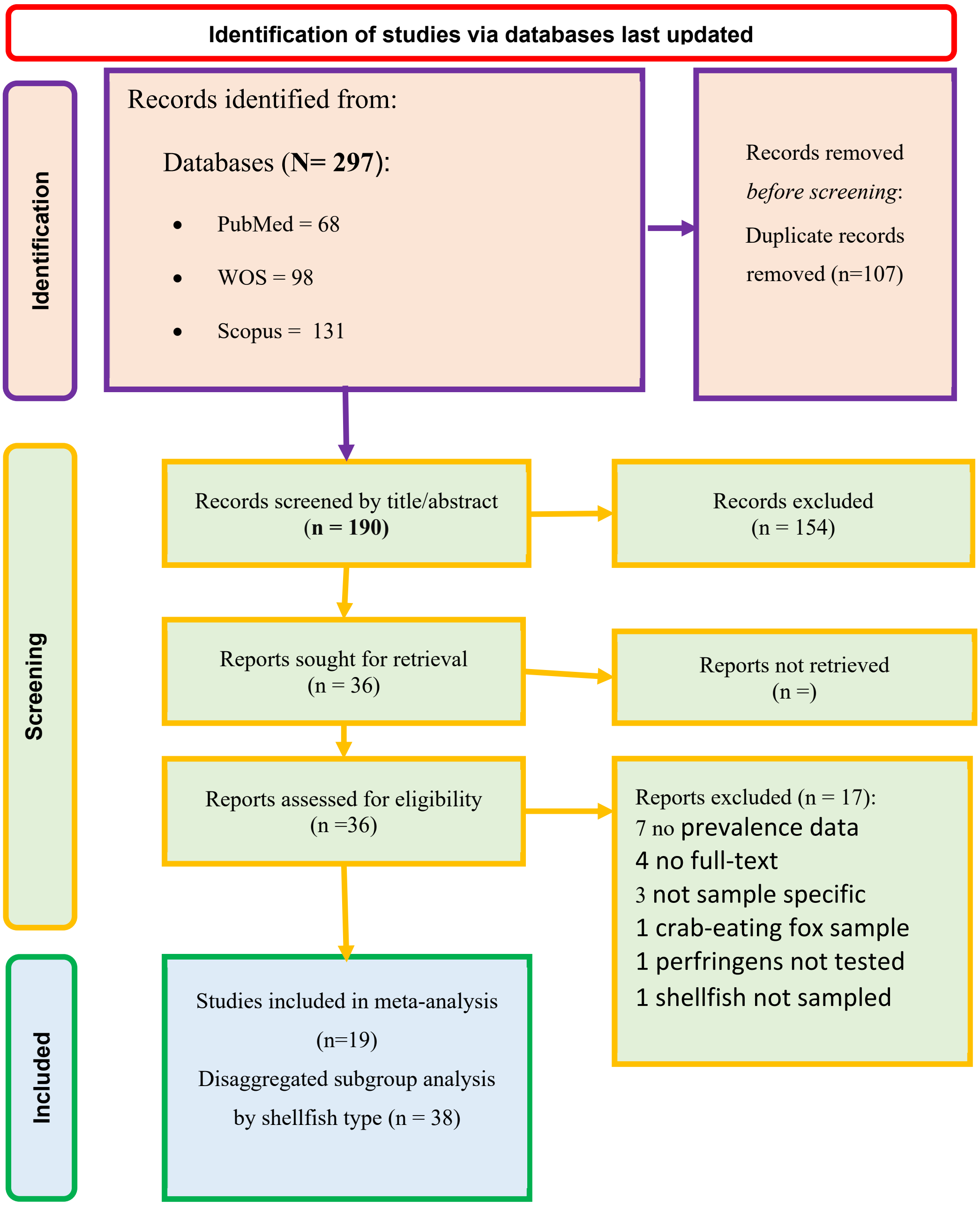

Figure S1. Flow diagram for processing data sources on shellfish-borne *C. perfringens*. #additional article from review of selected studies reference list.

A: Overall

Study	Events	Total	Events per 100 observations GLMM, Random, 95% CI
Ohnish et al. 2025 (C. perfringens; Molluscs (Shellfish); Japan)	44	65	67.69 [54.95; 78.77]
Li et al. 2024 (C. perfringens; Molluscs (clams/oysters/snails); China)	46	74	62.16 [50.13; 73.19]
Yanagimoto et al. 2020 (C. perfringens; Molluscs (clams); Japan)	7	76	9.21 [3.78; 18.06]
Regan et al. 2018 (C. perfringens; Molluscs (shellfish/squid); USA)	2	36	5.56 [0.68; 18.66]
Lopez-Mendoza et al. 2016 (C. perfringens; Molluscs (mussels); Spain)	13	15	86.67 [59.54; 98.34]
La Sala et al. 2015 (C. perfringens; Crustacean (crabs); Argentina)	54	110	49.09 [39.43; 58.80]
Ates et al. 2011 (C. perfringens; Molluscs (mussels); Turkey)	600	600	100.00 [99.39; 100.00]
Gonzalez et al. 2011 (C. perfringens; Molluscs (oyster); Venezuela)	172	330	52.12 [46.58; 57.62]
Papadopoulou et al. 2007 (C. perfringens; Crustacean (prawns)/Molluscs (squid/octopus/mussels); Greece)	0	155	0.00 [0.00; 2.35]
Miller et al. 2006 (C. perfringens; Molluscs(clams/mussels/ Crustacean (crabs); USA)	32	59	54.24 [40.75; 67.28]
Muniain-Mujika et al. 2003 (C. perfringens; Molluscs(mussels/oysters); Spain)	48	60	80.00 [67.67; 89.22]
Saito, 1990 (C. perfringens; Molluscs (oysters); Japan)	41	41	100.00 [91.40; 100.00]
Oka et al. 1989 (C. perfringens; Molluscs (squid/Scallop/clams); Japan)	15	40	37.50 [22.73; 54.20]
Madden et al. 1986 (C. perfringens; Molluscs (mussels); UK)	279	288	96.88 [94.15; 98.56]
Abeyta, 1983 (C. perfringens; Crustacean (Crab /Shrimp)/Molluscs (Oyster /Clam/Scallop/Mussel/Squid) ; USA)	6	132	4.55 [1.69; 9.63]
Christopher et al. 1978 (C. perfringens; Crustacean (shrimps); USA)	0	60	0.00 [0.00; 5.96]
Fruin, 1978 (C. perfringens; Crustacean (crab)/Molluscs (clams); USA)	29	33	87.88 [71.80; 96.60]
Inal et al. 1974 (C. perfringens; Molluscs (mussels); Germany)	71	147	48.30 [39.99; 56.68]
Elliott, 1970 (C. perfringens; Molluscs (slug); USA)	10	15	66.67 [38.38; 88.18]
**Total (95% CI)**		**2336**	**54.12 [19.73; 84.99]**
**95% Prediction interval**			**[0.08; 99.94]**

Heterogeneity: $Tau^2 = 11.4596$; $Chi^2 = 253.80$, df = 18 ($P < 0.0001$); $I^2 = 92.9\%$ [90.3%; 94.8%]

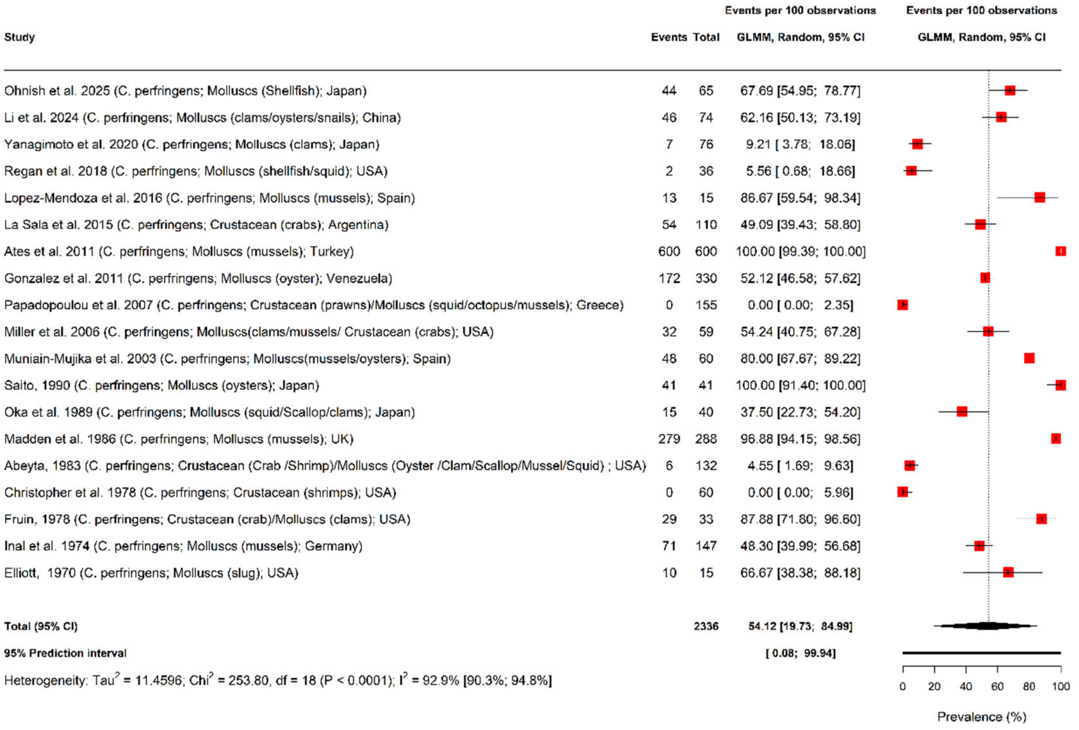

B: Toxigenic

Study	Events	Total	Events per 100 observations GLMM, Random, 95% CI
Ohnish et al. 2025 (C. perfringens; Molluscs (Shellfish); Japan)	44	65	67.69 [54.95; 78.77]
Li et al. 2024 (C. perfringens; Molluscs (clams/oysters/snails); China)	46	74	62.16 [50.13; 73.19]
Yanagimoto et al. 2020 (C. perfringens; Molluscs (clams); Japan)	7	76	9.21 [3.78; 18.06]
Regan et al. 2018 (C. perfringens; Molluscs (shellfish/squid); USA)	2	36	5.56 [0.68; 18.66]
Lopez-Mendoza et al. 2016 (C. perfringens; Molluscs (mussels); Spain)	.	15	
La Sala et al. 2015 (C. perfringens; Crustacean (crabs); Argentina)	54	110	49.09 [39.43; 58.80]
Ates et al. 2011 (C. perfringens; Molluscs (mussels); Turkey)	.	600	
Gonzalez et al. 2011 (C. perfringens; Molluscs (oyster); Venezuela)	.	330	
Papadopoulou et al. 2007 (C. perfringens; Crustacean (prawns)/Molluscs (squid/octopus/mussels); Greece)	.	155	
Miller et al. 2006 (C. perfringens; Molluscs(clams/mussels/ Crustacean (crabs); USA)	.	59	
Muniain-Mujika et al. 2003 (C. perfringens; Molluscs(mussels/oysters); Spain)	.	60	
Saito, 1990 (C. perfringens; Molluscs (oysters); Japan)	9	41	21.95 [10.56; 37.61]
Oka et al. 1989 (C. perfringens; Molluscs (squid/Scallop/clams); Japan)	4	40	10.00 [2.79; 23.66]
Madden et al. 1986 (C. perfringens; Molluscs (mussels); UK)	.	288	
Abeyta, 1983 (C. perfringens; Crustacean (Crab /Shrimp)/Molluscs (Oyster /Clam/Scallop/Mussel/Squid) ; USA)	.	132	
Christopher et al. 1978 (C. perfringens; Crustacean (shrimps); USA)	.	60	
Fruin, 1978 (C. perfringens; Crustacean (crab)/Molluscs (clams); USA)	25	33	75.76 [57.74; 88.91]
Inal et al. 1974 (C. perfringens; Molluscs (mussels); Germany)	.	147	
Elliott, 1970 (C. perfringens; Molluscs (slug); USA)	.	15	
**Total (95% CI)**		**2336**	**32.01 [14.51; 56.63]**
**95% Prediction interval**			**[1.31; 94.33]**

Heterogeneity: $Tau^2 = 2.0026$; $Chi^2 = 95.48$, df = 7 ($P < 0.0001$); $I^2 = 92.7\%$ [87.9%; 95.6%]

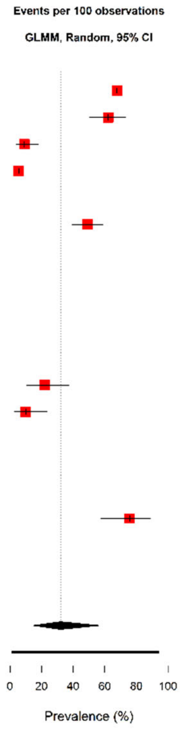

Figure S2: Detailed prevalence of shellfish-borne *C. perfringens*.

A: Era

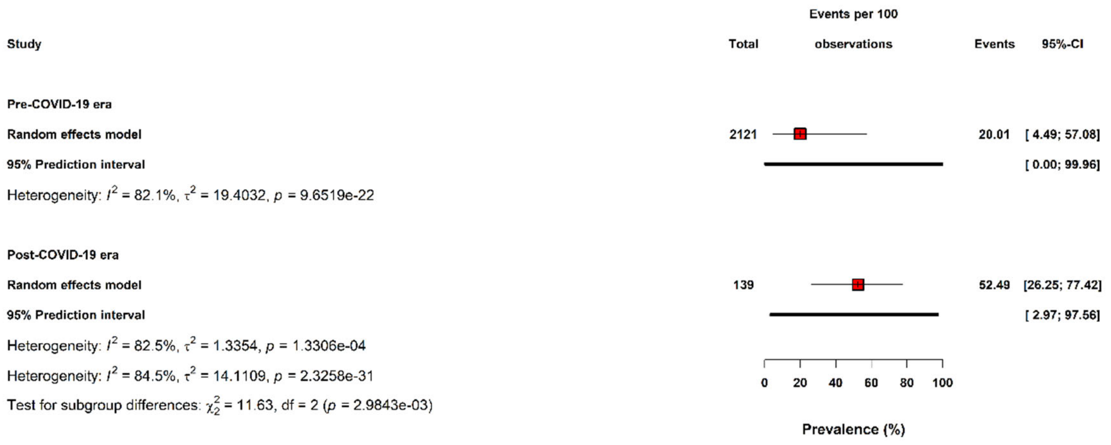

B: Period

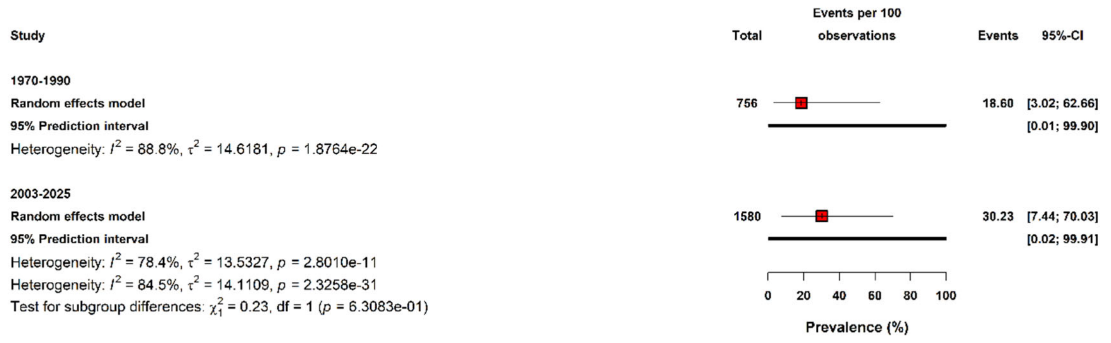

Figure S3 Detailed prevalence of shellfish-borne C. perfringens by temporal patterns

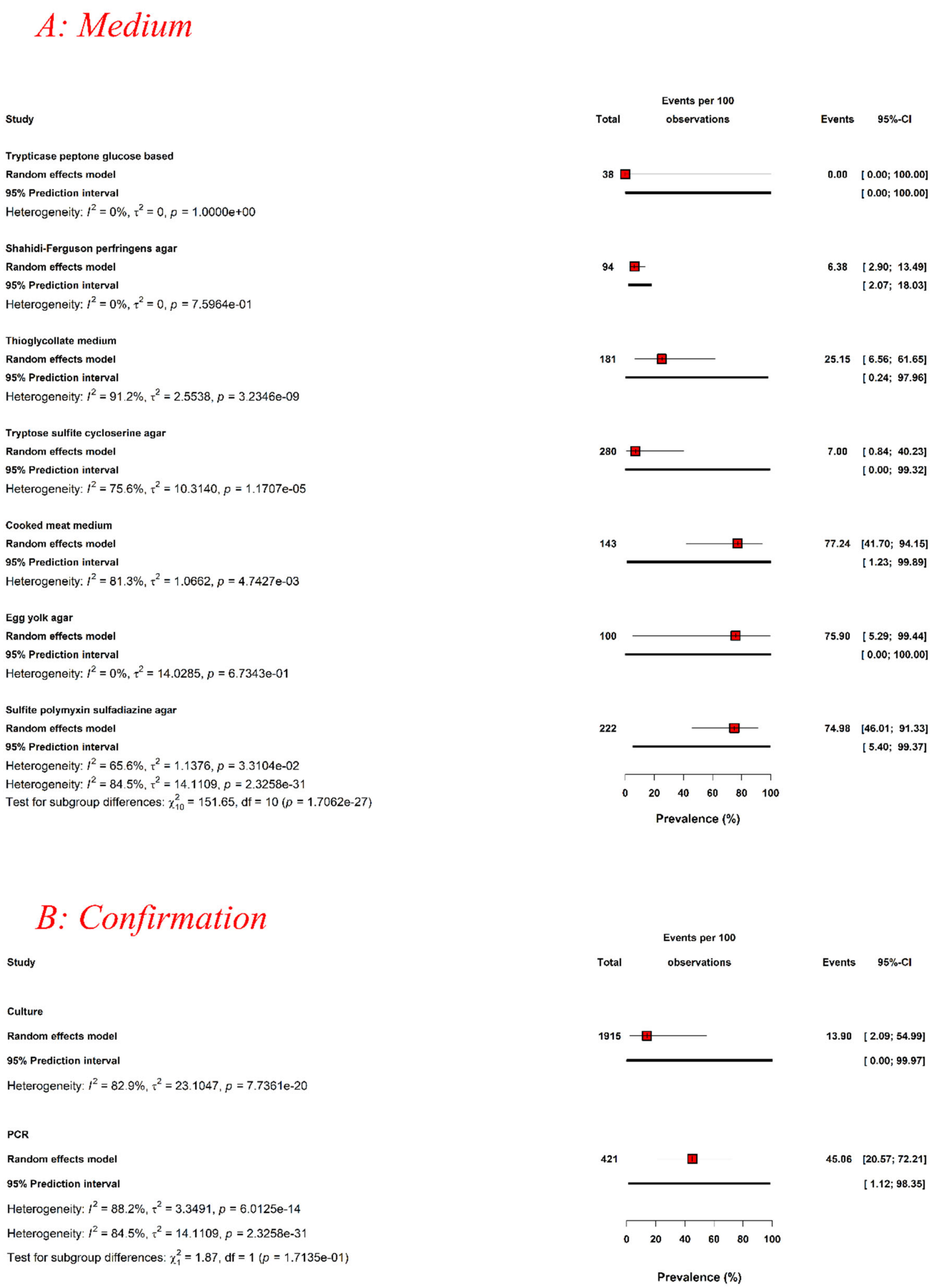

Figure S4: Detailed pooled prevalence of shellfish-borne *C. perfringens* by growth media and confirmation method.

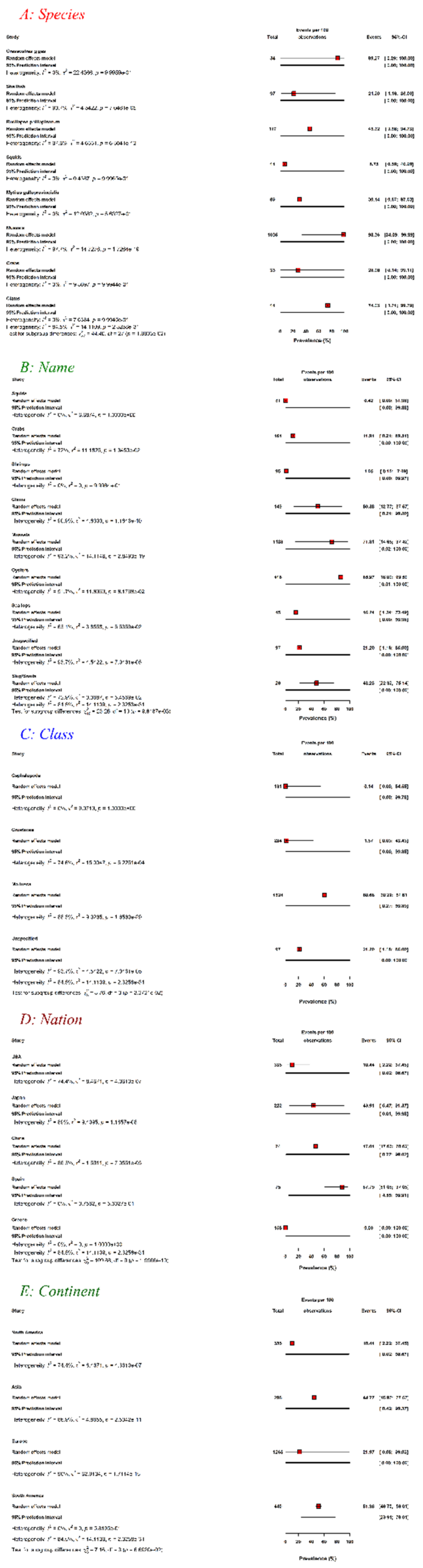
A: Species
B: Name
C: Class
D: Nation
E: Continent

Figure S5 Detailed shellfish-borne *C. perfringens* prevalence by shellfish scientific names, common name, scientific class, nation, and continents.

Table S1: Full coefficients of the various variables in the meta-regression models.

Model's variables	estimate	se	ci.lb	ci.ub	pval
**Medium**					
Intercept	1.0819	1.0187	-1.0083	3.1721	0.2976
Reference: Cooked meat medium					
Differential reinforced clostridial broth	6.009	2.5158	0.8469	11.1711	0.0242
Egg yolk agar	-0.1842	1.4208	-3.0994	2.731	0.8978
Iron milk	-0.997	1.8365	-4.7653	2.7712	0.5917
Oleandomycin polymyxin sulphadiazine agar	2.3521	1.8776	-1.5004	6.2045	0.221
Shahidi-Ferguson perfringens agar	-3.6348	1.3375	-6.3791	-0.8906	0.0113
Sulfite polymyxin sulfadiazine agar	-0.1603	1.3287	-2.8867	2.566	0.9049
Thioglycollate	-1.8848	1.2785	-4.5081	0.7385	0.152
Trypticase peptone glucose based	-4.7403	1.929	-8.6982	-0.7825	0.0207
Trypticase soy agar	-5.8777	2.5202	-11.0488	-0.7067	0.0274
Tryptose sulfite cycloserine agar	-2.4184	1.1745	-4.8282	-0.0086	0.0492
**Continent**					
Intercept	-0.1849	0.8283	-1.8683	1.4985	0.8247
Reference: Asia					
Europe	0.3293	1.1999	-2.1092	2.7678	0.7854
North America	-1.3006	1.076	-3.4873	0.8861	0.2351
South America	0.2093	1.9392	-3.7315	4.1502	0.9147
**Nation**					
Intercept	-0.0364	1.6433	-3.4024	3.3297	0.9825
Reference: Argentina					
China	-0.0578	1.8734	-3.8953	3.7798	0.9756
Germany	-0.0317	2.3211	-4.7862	4.7228	0.9892
Greece	-4.2825	2.024	-8.4285	-0.1366	0.0434
Japan	-0.2318	1.8148	-3.9494	3.4857	0.8993
Spain	1.8738	1.9798	-2.1817	5.9293	0.352
Turkey	7.1273	2.8718	1.2446	13.0099	0.0193
United Kingdom	3.4704	2.3482	-1.3397	8.2804	0.1506
USA	-1.3091	1.7213	-4.8351	2.2169	0.4533
Venezuela	0.1213	2.3164	-4.6236	4.8661	0.9586
**Shellfish types**					
Intercept	-0.9808	2.7519	-7.1124	5.1508	0.7289
reference: Bullacta exerata Philippi					
Cancer magister	-2.7804	4.6173	-13.0683	7.5076	0.5605
Clams	0.8952	3.5791	-7.0796	8.8699	0.8076
Corbicula fluminea,Tresus nuttalli, Saxidomus nuttalli, Macoma spp.	2.5903	4.246	-6.8705	12.051	0.5554
Crabs	1.5074	3.4999	-6.2909	9.3057	0.6758
Crassostrea gigas	0.9439	3.5537	-6.9741	8.8619	0.7959
Limax maximus	1.674	3.8119	-6.8194	10.1674	0.6699

Loligo opalescen	-2.5745	4.6218	-12.8724	7.7234	0.5897
Loligo vulgaris	-3.6343	4.606	-13.8972	6.6286	0.4484
Mussels	3.611	3.1703	-3.4528	10.6749	0.2812
Mytilus edulis	-0.891	3.9506	-9.6934	7.9114	0.8261
Mytilus galloprovincialis	1.2324	3.2199	-5.9418	8.4067	0.7099
Mytilus galloprovincialis; Mytilus spp	1.1856	3.6993	-7.057	9.4282	0.7552
Neohelice granulata/Cyrtograpsus angulatus	0.9445	3.675	-7.244	9.1329	0.8024
Octopus vulgaris	-3.6343	4.606	-13.8972	6.6286	0.4484
Ostreidae	0.7985	3.8457	-7.7703	9.3673	0.8397
Oyster Crassostrea rhizophorae	1.0657	3.6622	-7.0941	9.2255	0.777
Oysters	5.3997	4.6078	-4.8672	15.6666	0.2684
Pandalus jordani	-2.5455	4.1672	-11.8307	6.7396	0.5549
Patinopecten caurinus	-2.3865	4.6267	-12.6955	7.9225	0.6172
Pectinidae	-0.5232	3.9673	-9.363	8.3165	0.8977
Penaeus kerathurus	-2.951	4.6142	-13.2321	7.3301	0.5368
Penaeus stylirostris, Penaeus vannamei, and Penaeus setiferus	-3.815	4.6047	-14.0748	6.4448	0.4267
Ruditapes philippinarum	0.7792	3.2926	-6.5573	8.1156	0.8177
Saxidomus giganteus	-1.4171	4.1958	-10.7659	7.9318	0.7425
Scallops	1.5999	3.7708	-6.8019	10.0016	0.6803
Shellfish	0.1254	3.3645	-7.3712	7.622	0.971
Squids	-0.9751	3.703	-9.2259	7.2758	0.7977
**Class**					
Intercept	-3.329	1.1783	-5.7236	-0.9344	0.0079
Reference: Cephalopoda					
Crustacea	1.3041	1.5067	-1.7578	4.3661	0.3928
Mollusca	3.6149	1.2728	1.0283	6.2015	0.0076
Unspecified	2.2012	2.0109	-1.8855	6.288	0.2814
**Name**					
Intercept	-0.1735	0.9891	-2.2029	1.8559	0.862
Reference: Clams					
Crabs	-0.4439	1.5767	-3.6789	2.7912	0.7805
Mussels	1.0886	1.2898	-1.5579	3.7352	0.4061
Octopus	-4.4416	2.9436	-10.4814	1.5982	0.1429
Oysters	0.9183	1.4694	-2.0966	3.9331	0.5373
Prawns	-3.7583	2.9486	-9.8083	2.2917	0.2133
Scallops	-0.9655	1.7156	-4.4857	2.5546	0.5782
Shrimps	-3.9198	2.0998	-8.2283	0.3887	0.0728
Slug/Snails	0.0487	1.8945	-3.8386	3.936	0.9797
Squids	-2.83	1.6847	-6.2867	0.6266	0.1045
Unspecified	-0.899	1.9163	-4.8309	3.033	0.6428
